# Real-world effectiveness and prognostic factors of durvalumab plus chemotherapy in a multicentric cohort with advanced biliary tract cancer

**DOI:** 10.1093/oncolo/oyae306

**Published:** 2024-11-20

**Authors:** Wen-Kuan Huang, Yan-Jei Tang, Chiao-En Wu, Ming-Mo Hou, Hung-Chih Hsu, Po-Jung Su, Nai-Jung Chiang, San-Chi Chen, Chun-Nan Yeh, Jen-Shi Chen, Ming-Huang Chen, Chia-Hsun Hsieh, Wen-Chi Chou

**Affiliations:** Division of Hematology-Oncology, Department of Internal Medicine, Chang Gung Memorial Hospital at Linkou, Chang Gung University College of Medicine, Taoyuan, Taiwan; Division of Hematology-Oncology, Department of Internal Medicine, Chang Gung Memorial Hospital at Linkou, Chang Gung University College of Medicine, Taoyuan, Taiwan; Division of Hematology-Oncology, Department of Internal Medicine, Chang Gung Memorial Hospital at Linkou, Chang Gung University College of Medicine, Taoyuan, Taiwan; Division of Hematology-Oncology, Department of Internal Medicine, Chang Gung Memorial Hospital at Linkou, Chang Gung University College of Medicine, Taoyuan, Taiwan; Division of Hematology-Oncology, Department of Internal Medicine, Chang Gung Memorial Hospital at Linkou, Chang Gung University College of Medicine, Taoyuan, Taiwan; Division of Hematology-Oncology, Department of Internal Medicine, Chang Gung Memorial Hospital at Linkou, Chang Gung University College of Medicine, Taoyuan, Taiwan; Department of Oncology, Taipei Veterans General Hospital, Taipei, Taiwan; School of Medicine, National Yang Ming Chiao Tung University, Taipei, Taiwan; Department of Oncology, Taipei Veterans General Hospital, Taipei, Taiwan; School of Medicine, National Yang Ming Chiao Tung University, Taipei, Taiwan; Department of General Surgery, GIST Team, and Liver Research Center, Chang Gung Memorial Hospital, Linkou Branch, Chang Gung University, Taoyuan, Taiwan; Division of Hematology-Oncology, Department of Internal Medicine, Chang Gung Memorial Hospital at Linkou, Chang Gung University College of Medicine, Taoyuan, Taiwan; Department of Oncology, Taipei Veterans General Hospital, Taipei, Taiwan; School of Medicine, National Yang Ming Chiao Tung University, Taipei, Taiwan; Division of Hematology-Oncology, Department of Internal Medicine, Chang Gung Memorial Hospital at Linkou, Chang Gung University College of Medicine, Taoyuan, Taiwan; Division of Hematology-Oncology, Department of Internal Medicine, New Taipei City Municipal Tucheng Hospital, New Taipei City, Taiwan; Division of Hematology-Oncology, Department of Internal Medicine, Chang Gung Memorial Hospital at Linkou, Chang Gung University College of Medicine, Taoyuan, Taiwan

**Keywords:** biliary tract cancer, multicenter analysis, durvalumab dose-response relationship, prognostic factors

## Abstract

**Background:**

Biliary tract cancer (BTC) is an aggressive biliary tract cancer, arising from the bile ducts and gallbladder, with a poor prognosis. The TOPAZ-1 trial of durvalumab plus first-line chemotherapy (gemcitabine plus cisplatin) showed improved survival vs chemotherapy alone. This real-world study aimed to confirm the effectiveness of this regimen.

**Methods:**

This retrospective, multicenter study included patients with advanced BTC treated with first-line durvalumab plus platinum chemotherapy at the Linkou, Taoyuan, and Tucheng branches of Chang Gung Memorial Hospital as well as at Taipei Veterans General Hospital between August 2021 and June 2023.

**Results:**

Among the 45 patients with advanced biliary tree cancer treated with durvalumab plus cisplatin and gemcitabine as first-line treatment, the objective response rate was 31.1% (14 partial responses). An additional 40% (18 patients) had stable disease. The median progression-free survival was 5.6 months (95%CI, 4.4-6.9) and median overall survival was 15.8 months (95%CI, 7.9-23.8). Responders had significantly longer survival than non-responders (15.8 vs 3.3 months). Although higher durvalumab doses (1000-1500 mg) appeared to have improved efficacy compared to lower doses (<1000 mg), the difference was not statistically significant. On multivariate analysis, poor ECOG performance status (≥2) and a high neutrophil-lymphocyte ratio were independent prognostic factors for shorter overall survival.

**Conclusion:**

This real-world study demonstrated the comparable efficacy of durvalumab plus chemotherapy to the TOPAZ-1 trial for patients with advanced BTC and identified prognostic factors. There was a trend toward improved efficacy with higher durvalumab dosing (1000-1500 mg) vs lower dosing, though further research is needed to confirm this relationship.

**Implications for practice**

This real-world study demonstrated the comparable efficacy of durvalumab plus chemotherapy to the TOPAZ-1 trial for patients with advanced BTC and identified prognostic factors. Improved efficacy seems to be associated with higher durvalumab dosing (1000-1500 mg) vs lower dosing. These findings may help guide clinical decision-making and provide important insights for future prospective studies in this patient population.

## Introduction

Biliary tract cancer (BTC) is an uncommon and highly aggressive cancer arising from the epithelial cells of the bile ducts. Most patients are diagnosed at an advanced stage when treatment options are limited, contributing to an extremely poor prognosis with 5-year survival rates of only 7%-20%.^1^ For these patients with unresectable or metastatic disease, cisplatin plus gemcitabine chemotherapy is the mainstay of treatment and prognosis remains poor with a median survival of 9-12 months.^2-4^

BTCs are notable for their desmoplastic and fibroinflammatory microenvironment which exhibits a poorly immunogenic niche characterized by several immunosuppressive cell types.^5^ Early clinical investigations demonstrated that while these immunotherapies led to durable antitumor responses in a subset of BTC cases, overall response rates have remained relatively low.^6-8^ However, these immune checkpoint inhibitors were well-tolerated and confirmed to have antitumor capability. This has now led to multiple efforts assessing combinations of immune checkpoint blockade with chemotherapy as first-line regimens for patients with advanced-stage biliary tract cancer.^9^ The phase 3 TOPAZ-1 trial recently demonstrated significantly improved overall survival (OS; median, 12.8 vs 11.5 months, HR = 0.8, *P* = .021) with the addition of the PD-L1 inhibitor durvalumab to standard first-line chemotherapy (gemcitabine plus cisplatin) compared to chemotherapy alone in patients with advanced biliary tract cancer.^10^ Consequently, the results of TOPAZ-1 led to the FDA and EMA approval of durvalumab plus chemotherapy as the new first-line standard treatment for patients with advanced biliary tract cancer.

While encouraging, the TOPAZ-1 trial enrolled a highly selected patient population that may not reflect real-world clinical scenarios. In this multicenter real-world study, we aimed to confirm the effectiveness of this emerging chemoimmunotherapy regimen for broader unresectable or metastatic BTC and identify prognostic factors associated with survival with first-line durvalumab plus gemcitabine and cisplatin.

## Patients and methods

### Study design and patients

In this multicenter study, we retrospectively collected data on patients with unresectable advanced or recurrent biliary tract cancer who were treated with durvalumab. Patients were registered between August 2021 and June 2023 at the Linkou, Taoyuan, and Tucheng branches of Chang Gung Memorial Hospital (CGMH) as well as Taipei Veterans General Hospital (TVGH). These registered patients were observed until the cutoff date for the follow-up (December 2023) or death after initiating durvalumab treatment. Fifty-three patients were identified, of which 8 patients receiving second or third-line durvalumab treatment were excluded. The remaining 45 patients were included in the analysis, with 5 study participants recruited from TVGH and 40 participants selected from CGMH branches (Supplementary Figure S1). This study was reviewed and approved by the Institutional Review Board of Chang Gung Memorial Hospital (IRB No. 202302254B0).

### Treatment

Patients were treated with the durvalumab plus platinum-based chemotherapy. The standard dosage of durvalumab is 1500 mg every 3 weeks. However, as this medication is not fully covered by Taiwan’s National Health Insurance program, patients must pay out-of-pocket. Therefore, the dosage can vary depending on each patient’s financial situation. In this real-world analysis, we will examine the correlation between durvalumab dosage and anti-tumor efficacy.

### Study endpoints and assessment

The main study endpoints included OS, progression-free survival (PFS), and best overall response rate (ORR). The OS was defined as the time from the first durvalumab combination therapy to the date of death due to any cause, or was censored on the day of cutoff. The PFS was defined as the time from the start of the first durvalumab combination therapy to the date of disease progression according to radiographic assessment, death due to any cause, or was censored on the day of cutoff, whichever came first.

In these real-world settings, each patient generally underwent tumor imaging assessment every 9-12 weeks using magnetic resonance imaging (MRI) or computed tomography (CT) scans. Tumor response was evaluated locally based on the response evaluation criteria in solid tumors version 1.1. The disease control rate (DCR) was defined as the percentage of patients displaying stable disease (SD), partial response (PR), or complete response (CR).

### Data collection

The patient demographic and clinical characteristics were retrospectively reviewed using electronic medical records from participating institutions. Data on performance status, primary tumor site, viral hepatitis status, tumor characteristics, tumor markers, durvalumab dose, and neutrophil-to-lymphocyte ratios (NLRs) were obtained.

### Statistical analysis

Data of categorical variables were expressed as frequency counts and percentages, continuous variables as mean and standard deviation, and those apparently skewed continuous variables (eg, NLRs) as median with interquartile range (IQR), respectively. The median PFS and OS were estimated using the Kaplan-Meier method. The OS of patients by treatment response (PR, SD and progressive disease [PD]) or combined positive score (CPS <1 vs CPS ≥1) of programmed cell death-ligand 1 (PD-L1) was compared using the log-rank test. Univariate Cox proportional hazard analysis was performed to evaluate the potential associated factors of PFS and OS, including durvalumab treatment dose (ie, ≥1400 mg [median] vs <1400 mg and per 100 mg increase), age, sex, Eastern Cooperative Oncology Group (ECOG) performance status, and primary site, disease status, disease classification, viral status, baseline carbohydrate antigen (CA) 19-9 (≥92.9 mg/ml [median] vs < 92.9 mg/mL), and NLRs (≥4.24 [median] vs<4.24). Currently there is no consensus regarding the cutoffs for Ca19-9 and NLR in biliary tract cancer literature, therefore we chose to divided the patients into 2 equally sized groups by the median value. Multivariable Cox analysis was performed to identify the independent prognostic factors by incorporating the variables with borderline significance or significance (*P* < .1) in the univariate Cox analyses. It is noted that CPS of PD-L1 was not included in the risk factor analysis since nearly half of the data were not available. In an additional analysis, we manipulated the durvalumab treatment dose as a restricted cubic spline (RCS) variable with 3 default knots to explore the relationship between dose and outcomes of interest, including treatment response (PR or CR), PD, PFS, and OS. A 2-sided *P* value less than .05 was statistically significant. RCS modeling was performed with R version 4.3.2 (R Project for Statistical Computing) and the package “rms” version 6.7-1.^11^ Other analyses were carried out using SPSS 26 (IBM SPSS Inc.).

## Results

### Patient and disease characteristics

A total of 45 patients treated with first-line treatment with durvalumab plus platinum-based chemotherapy were included. The baseline demographics and disease characteristics of the patients along with durvalumab doses administered to each patient with advanced biliary tract cancer in the CGMH cohort are summarized in Table 1 and Supplementary Figure S2. The cohort had a median (range) age of 59 years (36-89) and was comprised of 20 men (44.4%) and 25 women (55.6%). Two patients received oxaliplatin and gemcitabine, the other patients received cisplatin and Gemcitabine. Only 1 patient received palliative radiation and 4 patients underwent liver-directed therapy. In terms of disease status, 41 (91.1%) patients had initially unresectable disease while 4 (8.9%) had recurrent disease after prior resection. The majority (*n* = 34, 75.6%) presented with metastatic disease, with the remaining 11 (24.4%) having locally advanced tumors. Any viral hepatitis infection was present in 15 (33.3%) patients. The median durvalumab dose received was 1440 mg (IQR 1000-1440 mg). The median follow-up duration was 7.9 months [IQR 4.7-12.2 months]. A total of 35 (77%) patients had discontinued first-line treatment.

**Table 1.** Demographics and characteristics of patients.

Variable	Statistics
Age, year	59.9 ± 11.1
Male sex	20 (44.4)
ECOG performance status	
0	12 (26.7)
1	22 (48.9)
2	4 (8.9)
3	5 (11.1)
4	2 (4.4)
Site	
Gallbladder	7 (15.6)
Intrahepatic	31 (68.9)
Extrahepatic	7 (15.6)
Disease status	
Recurrent	4 (8.9)
Initially unresectable	41 (91.1)
Disease classification	
Locally advanced	11 (24.4)
Metastatic	34 (75.6)
Viral status	
No viral hepatitis	30 (66.7)
Hepatitis B	12 (26.7)
Hepatitis C	3 (6.7)
Durvalumab dose, mg	1440 [1000, 1440]
CEA before CT	4.2 [1.8, 9.5]
CA 19-9 before CT	93 [12, 3629]
Neutrophil-to-lymphocyte ratio	4.2 [3.0, 5.5]
<Median	22 (48.9)
≥Median	23 (51.1)
PD-L1	
Negative (CPS < 1)	18 (75.0)
Positive (CPS ≥ 1)	6 (25.0)
Missing data	21
Treatment response	
Complete response or partial response	14 (31.1)
Stable disease	18 (40.0)
Progression disease	13 (28.9)
Liver-targeted therapy	4 (8.9)
Palliative radiation	1 (2.2)
Follow-up time, month	
Mean ± standard deviation	8.4 ± 4.6
Median [25th, 75th percentile]	7.9 [4.7, 12.2]

Data are presented as frequency (%), mean ± SD or median [25th, 75th percentile].

Abbreviation: CA 19-9, carbohydrate antigen 19-9; CEA, carcinoembryonic antigen; CPS, combined positive score; CT, computed tomography; ECOG, eastern cooperative oncology group; PD-L1, programmed cell death-ligand 1.

### Treatment response and survival

**Figure 1.**
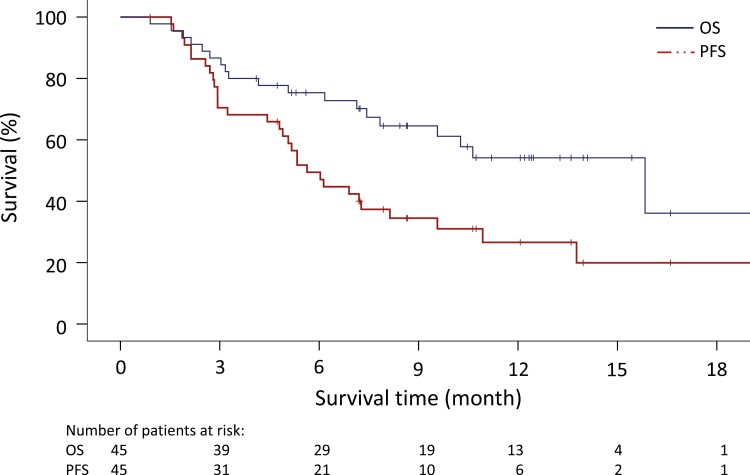
The Kaplan-Meier curves for progression free survival and overall survival. PFS, progressive free survival. Abbreviations: CI, confidence interval; CPS, combined positive score; NA, not applicable; OS, overall survival; PD, progression disease; PD-L1; programmed cell death-ligand 1; PR, partial response; SD, stable disease.

**Figure 2.**
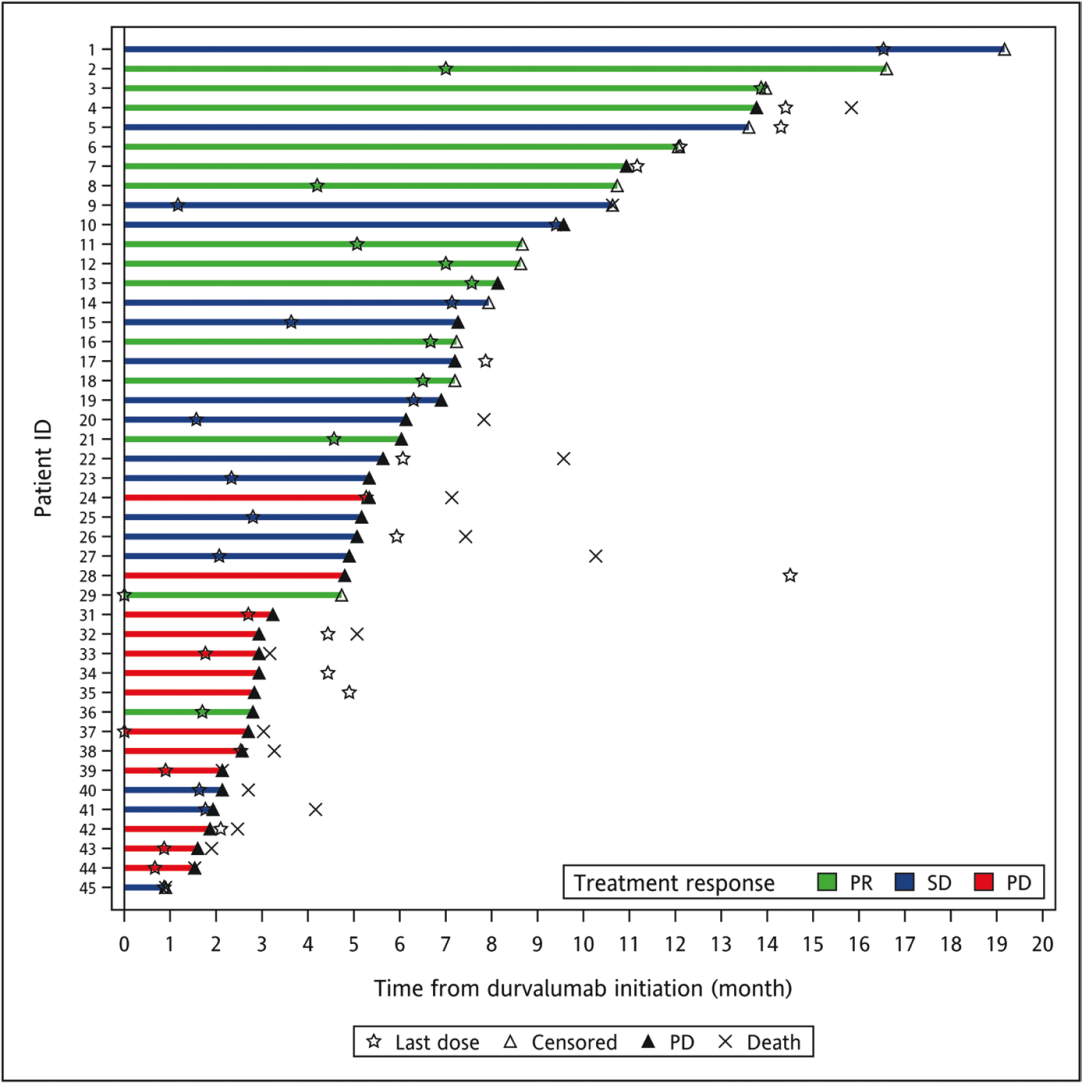
A swimmer plot depicting time to disease progression for patients receiving durvalumab plus chemotherapy. Each bar represents one subject in the study and their length of survival in months (*N* = 45). Abbreviations PD, progression disease; PR, partial response; SD, stable disease.

Of the 45 patients registered, 14 (31.1%) achieved an objective response defined as either CR or PR, with 1 patient achieving CR. Another 18 patients (40.0%) had a best response of SD. The remaining 13 patients (28.9%) demonstrated PD as their best response. ORR indicated the best response at any time compared to the baseline tumor burden from imaging studies such as CT/MRI scan. The overall ORR was 31.1% and the DCR was 71.1%. Median PFS was 5.6 months (95% confidence interval [CI], 4.4 to 6.9 months) and median OS was 15.8 months (95% CI, 7.9 to 23.8 months), as illustrated in Figure 1. At the time of data analysis, 31 patients (69%) developed disease progression and 19 patients (42%) had died. The OS rates at specified time points were 75.4% at 6 months, 54.1% at 12 months, and 36.1% at 18 months after initiating treatment. A swimmer plot depicting each patient’s best overall response, duration of durvalumab treatment, and time to progression is presented in Figure 2. Responders, who had either a partial response (PR) or stable disease (SD) for ≥6 months, had a median survival of 15.8 months. On the other hand, non-responders, who had stable disease for <6 months or progressive disease (PD), had a median survival of 3.3 months.

### Association between durvalumab dose and treatment efficacy

**Figure 3.**
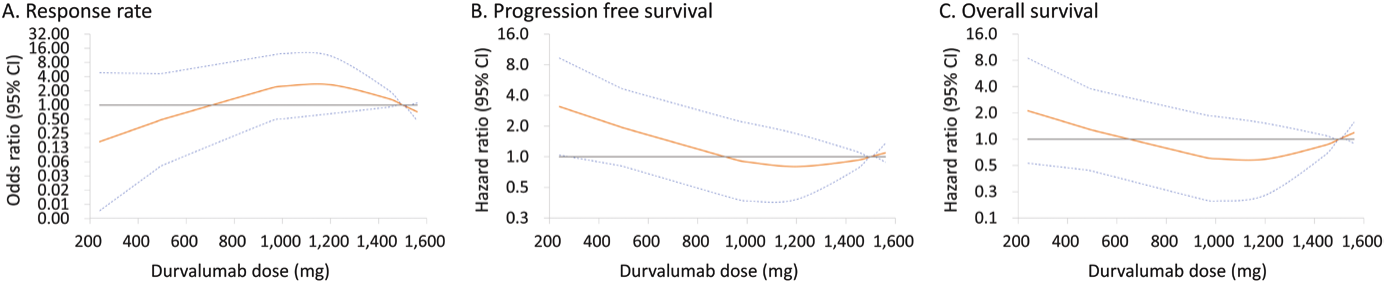
The restricted cubic spline (RCS) analysis revealed that the efficacy outcomes tended to be similar between doses of 1000 mg and 1500 mg but worsened at doses less than 1000 mg. The relationship between durvalumab dose and response rate (A), progression free survival (B), and overall survival (C) in which the durvalumab dose was treated as a restricted cubic spline variable with 3 knots. The dotted lines represent the 95% CIs.

The non-linear relationship between durvalumab dose and response rate, PFS, and OS based on the RCS method was explored (Figure 3). The RCS analysis suggested a trend where efficacy outcomes appeared similar between doses of 1000 mg and 1500 mg but potentially worsened at doses less than 1000 mg. However, these observations did not reach statistical significance, likely due to the limited sample size (*P* values for non-linearity >.05).

### Associate factors of progression free survival and overall survival

In this study, the multivariable Cox model indicated that ECOG performance status (PS)≥2 (hazard ratio [HR] 3.48, 95% CI, 1.44-8.41), CA 19-9 ≥ 92.9 U/mL before chemotherapy (HR 3.27, 95% CI, 1.44-7.42), and NLR ≥4.24 (HR 4.11, 95% CI, 1.76-9.58) were significantly associated with shorter PFS (Table 2, Figure S3). Moreover, the multivariable Cox model identified ECOG≥2 (HR 6.98, 95% CI: 2.29-21.29) and NLR ≥4.24 (HR 4.82, 95% CI, 1.63-14.20) as independent prognostic factors of OS.

**Table 2.** Associated factors of progression free survival and overall survival

Model / Variable	Progression free survival		Overall survival	
	Hazard ratio (95% CI)	*P* value	Hazard ratio (95% CI)	*P* value
Univariate analysis				
Durvalumab dose (≥1440 mg vs < 1440 mg)	0.85 (0.42-1.74)	.664	1.11 (0.45-2.75)	.819
Durvalumab dose per 100 mg	0.93 (0.84-1.02)	.115	0.97 (0.86-1.09)	.606
Age, year	1.02 (0.98-1.06)	.400	1.04 (0.99-1.09)	.127
Male	1.26 (0.62-2.56)	.520	1.80 (0.72-4.49)	.209
ECOG performance status (≥2 vs 0-1)	2.30 (1.05-5.06)	.038	4.14 (1.61-10.62)	.003
Site				
Intrahepatic vs Gallbladder	1.77 (0.61-5.16)	.292	0.83 (0.27-2.55)	.746
Extrahepatic vs gallbladder	0.87 (0.22-3.53)	.850	0.23 (0.02-2.05)	.187
Disease status (initially unresectable vs recurrent)	0.97 (0.29-3.20)	.954	1.89 (0.25-14.24)	.535
Disease classification (metastatic vs locally advanced)	1.15 (0.49-2.66)	.752	2.75 (0.63-11.92)	.178
Viral status (yes vs no)	1.27 (0.61-2.66)	.527	1.33 (0.52-3.40)	.545
CA 19-9 before CT (≥92.9 vs <92.9)	2.69 (1.28-5.65)	.009	2.72 (1.03-7.19)	.044
Neutrophil-to-lymphocyte ratio (≥ 4.24 vs <4.24)	2.26 (1.08-4.74)	.030	2.41 (0.91-6.36)	.076
Liver-targeted therapy	0.43 (0.10-1.83)	.255	NA	NA
Multivariable analysis				
ECOG performance status (≥2 vs 0-1)	3.48 (1.44-8.41)	.006	6.98 (2.29-21.29)	.001
CA 19-9 before CT (≥92.9 vs <92.9)	3.27 (1.44-7.42)	.005	2.33 (0.83-6.58)	.110
Neutrophil-to-lymphocyte ratio (≥4.24 vs <4.24)	4.11 (1.76-9.58)	.001	4.82 (1.63-14.20)	.004

Abbreviation: CA 19-9, carbohydrate antigen 19-9; CI, confidence Intervals; ECOG, eastern cooperative oncology group; NA, not applicable.

## Discussions

This multicenter, retrospective study confirmed the effectiveness of durvalumab plus chemotherapy in advanced biliary tract cancer in a real-world setting, compared to the TOPAZ-1 trial. Comparing with TOPAZ-1 trial, our real-world study had a smaller sample size (45 vs 685) and slightly younger patients (median age 59.9 vs 64). Notably, we included patients with poorer performance status (ECOG ≥ 2), a higher proportion of intrahepatic biliary tract cancer (68.8% vs 55.7%), and more initially unresectable disease (91.1% vs 80.4%), and viral hepatitis (33.5% vs 22.5%). The ORR was 31.1% and the DCR was 71.1% among 45 patients. The median PFS and OS of 5.6 and 15.8 months aligned with the pivotal trial. Efficacy outcomes trended better with higher (1000-1500 mg) vs lower durvalumab dosing. Poor performance status and high NLRs were independent poor prognostic factors for survival. Though limited by sample size, this study supports durvalumab plus chemotherapy as an effective first-line regimen in broader unresectable biliary tract cancer populations. Furthermore, the benefit of adding immunotherapy to chemotherapy for advanced biliary tract cancer has recently been confirmed by the phase III KEYNOTE-966 study, which combined pembrolizumab plus cisplatin and gemcitabine.^12^

Few real-world studies have investigated first-line immunotherapy plus chemotherapy for advanced biliary tract cancer. An Italian study demonstrated efficacy consistent with the phase III TOPAZ-1 trial, with a median PFS of 8.9 months, a median OS of 12.9 months, an ORR of 34.5%, and a DCR of 87.6% for durvalumab plus gemcitabine/cisplatin.^13^ Similarly, a China study found an ORR of 16.7%, a DCR of 79.6%, a median PFS of 6.6 months, and a median OS of 13.9 months across various PD-1 inhibitors combined with chemotherapy.^14^ Our study showed comparable efficacy in an Asian population, with a median OS of 15.8 months and an ORR of 31.1% for durvalumab plus chemotherapy. The shorter PFS observed in our study may be attributed to differences in the timing of imaging assessments and the evaluation for disease progression. These real-world results indicated clinically meaningful benefits of first-line immunotherapy combined with chemotherapy for a broader population of patients with advanced biliary tract cancer outside of clinical trials.

Retrospective analyses have shown that low-dose immune checkpoint inhibitors, such as pembrolizumab and nivolumab, demonstrate promising efficacy and safety profiles compared to standard dosing in various cancer types, including non-small cell lung cancer,^15^ hepatocellular carcinoma,^16^ renal cell carcinoma,^17^ and gynecologic cancers.^18^ In patients treated with low-dose regimens, such as pembrolizumab 100 mg every 3 weeks or nivolumab 20-100 mg every 2-3 weeks, response rates, PFS, and OS were comparable to those observed with standard dosing. Moreover, these low-dose approaches were well-tolerated, with similar safety profiles to standard dosing, and could potentially lead to substantial cost savings for healthcare systems. Our study observed a trend suggesting that lower doses of durvalumab might be associated with less favorable efficacy, though this relationship did not reach statistical significance. Of note, most of these low-dose immunotherapy studies involved immunotherapy as a single agent (monotherapy), whereas our study investigated the impact of combining immunotherapy and chemotherapy on anti-tumor efficacy. Further prospective randomized trials with sufficient sample sizes are warranted to compare the efficacy and safety of high-dose versus low-dose immune checkpoint inhibitors in combination with chemotherapy in patients with advanced biliary tract cancer.

This study identified poor PS (≥ 2) and high NLRs (≥ 4.24) as independent negative prognostic factors for PFS and OS in patients with advanced BTC receiving PD-1 inhibitors. These findings are complementary to the pivotal clinical trial, as patients with PS ≥ 2 and high NLRs were not included or well-represented in that trial. The association between high NLR and poor survival outcomes in our study is also consistent with previous reports in other cancer types, suggesting that systemic inflammation may play a role in modulating the efficacy of immunotherapy.^19,20^ To the best of our knowledge, this study is the first to demonstrate the prognostic value of the neutrophil-to-lymphocyte ratio (NLR) for patients treated with a combination of immunotherapy and chemotherapy. While a previous study in advanced BTC found that high carcinoembryonic antigen (CEA) levels were associated with poor survival outcomes,^14^ we found that high CA19-9 levels were independently associated with shorter PFS in our cohort. Taken together, our study provides insights into the prognostic factors associated with survival outcomes in a real-world population with advanced BTC receiving durvalumab plus chemotherapy.

While our results did not achieve statistical significance regarding the dose-outcome relationship, pharmacodynamic (PD) evidence indicates that a 10 mg/kg Q2W dose of durvalumab results in approximately 93.3% of patients achieving complete suppression of sPD-L1. Furthermore, pharmacokinetic (PK) analyses show that durvalumab exhibits a linear dose-response relationship at doses greater than 3 mg/kg Q2W, ensuring consistent and predictable drug exposure. In transitioning to a flat dosing regimen, this corresponds to approximately 15 mg/kg Q3W, translating to a required dose of around 900-975 mg for the average Taiwanese patient. These PD/PK findings suggested the importance of maintaining a dosage of at least 1000mg every three weeks (Q3W) to achieve clinical efficacy. Suboptimal dosing may result in reduced PD-L1 blockade and potentially inferior outcomes.

This study has several limitations that should be acknowledged. First, as a retrospective study, there is an inherent risk of bias and failure to collect information. For instance, nearly half of the patients lacked data on PD-L1 expression, which prevented the inclusion of this important biomarker in the risk factor analysis. Second, a limitation of this study is the potential impact of social determinants of health, such as socioeconomic status, access to healthcare and social support, which may confound the observed relationship between lower durvalumab dosing and unfavorable efficacy. Third, although the relationship between durvalumab dose and treatment efficacy was explored using RCS analysis, the limited sample size may have hindered the ability to detect statistically significant associations. Furthermore, the relatively short median follow-up duration of 7.9 months may not fully capture the long-term outcome. The results of this study may have limited external validity as they represent a select population from Taiwan. Factors such as viral hepatitis prevalence may differ in other populations. Finally, while several prognostic factors associated with survival outcomes were identified, such as ECOG PS, CA 19-9 levels, and NLRs, these findings need to be validated in large prospective studies to confirm their clinical utility in guiding treatment decisions.

In conclusion, this retrospective study provides real-world evidence on the efficacy of durvalumab plus platinum-based chemotherapy in patients with advanced biliary tract cancer, demonstrating comparable results to those observed in the pivotal clinical trial. Furthermore, we identified several independent prognostic factors, including ECOG PS ≥ 2, CA 19-9 ≥ 92.9 U/mL, and a neutrophil-to-lymphocyte ratio ≥ 4.24, which were significantly associated with unfavorable PFS and OS. These findings may help guide clinical decision-making and provide important insights for future prospective studies in this patient population.

## Supplementary Material

oyae306_suppl_Supplementary_Figures

## Data Availability

The data underlying this article will be shared on reasonable request to the corresponding author.
