# Supplementary material for: Real-world effectiveness and prognostic factors of durvalumab plus chemotherapy in a multicentric cohort with advanced biliary tract cancer
Source: Oncologist. 2024 Nov 20;30(8):oyae306. doi: 10.1093/oncolo/oyae306 (PMC12395238; doi:10.1093/oncolo/oyae306)
Supplement: oyae306_suppl_Supplementary_Figures [file oyae306_suppl_supplementary_figures.zip › Supplementary Figure/Supplementary Figure Captions.pdf]

Supplementary Figure Captions

Supplementary Figure S1. Patient Flow Diagram.

Supplementary Figure S2. Distribution of durvalumab doses administered to patients with advanced biliary tract cancer in the CGMH cohort (n = 45).

Supplementary Figure S3. The Kaplan-Meier curves for progression-free survival and overall survival stratified by ECOG status and the median value of NLR value.

Abbreviations: ECOG, Eastern Cooperative Oncology Group; NLR, neutrophil-to-lymphocyte ratio.
