# Supplementary figures and images for: Real-world effectiveness and prognostic factors of durvalumab plus chemotherapy in a multicentric cohort with advanced biliary tract cancer
Source: Oncologist. 2024 Nov 20;30(8):oyae306. doi: 10.1093/oncolo/oyae306 (PMC12395238; doi:10.1093/oncolo/oyae306)

## Slide 1
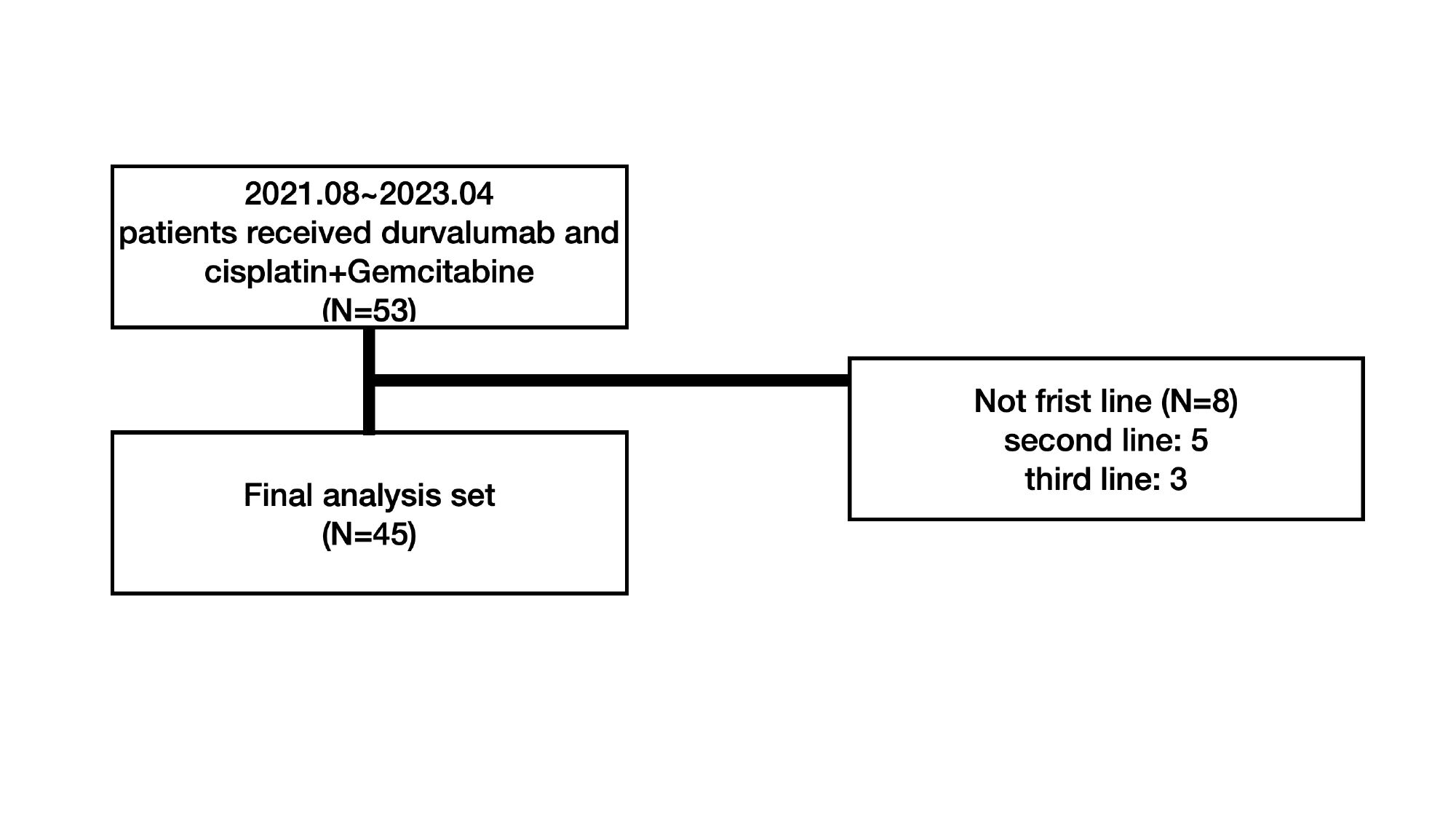

Supplement: oyae306_suppl_Supplementary_Figures [file oyae306_suppl_supplementary_figures.zip › Supplementary Figure/Figure S1.pptx]

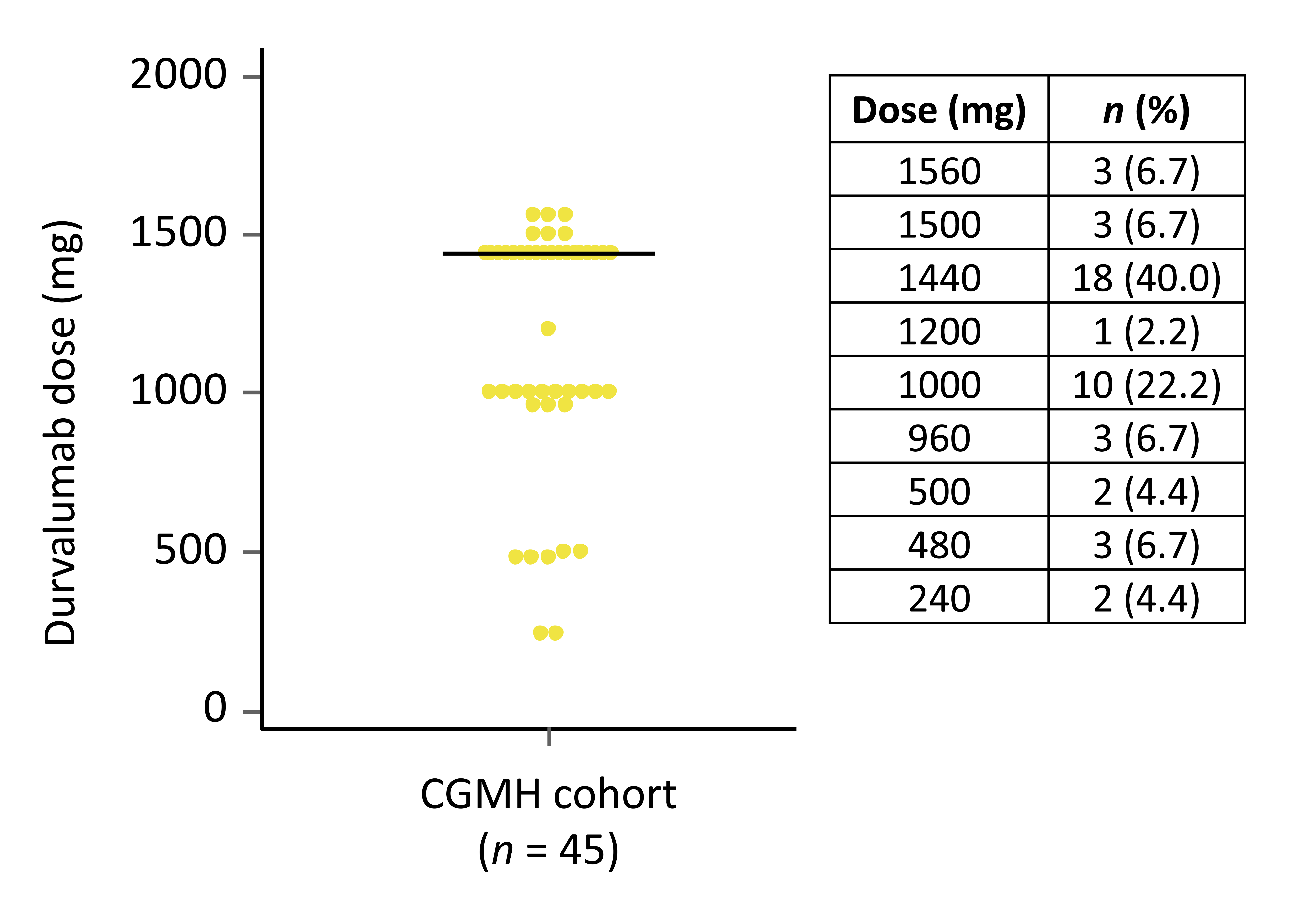

Supplement: oyae306_suppl_Supplementary_Figures [file oyae306_suppl_supplementary_figures.zip › Supplementary Figure/Figure S2.tif]

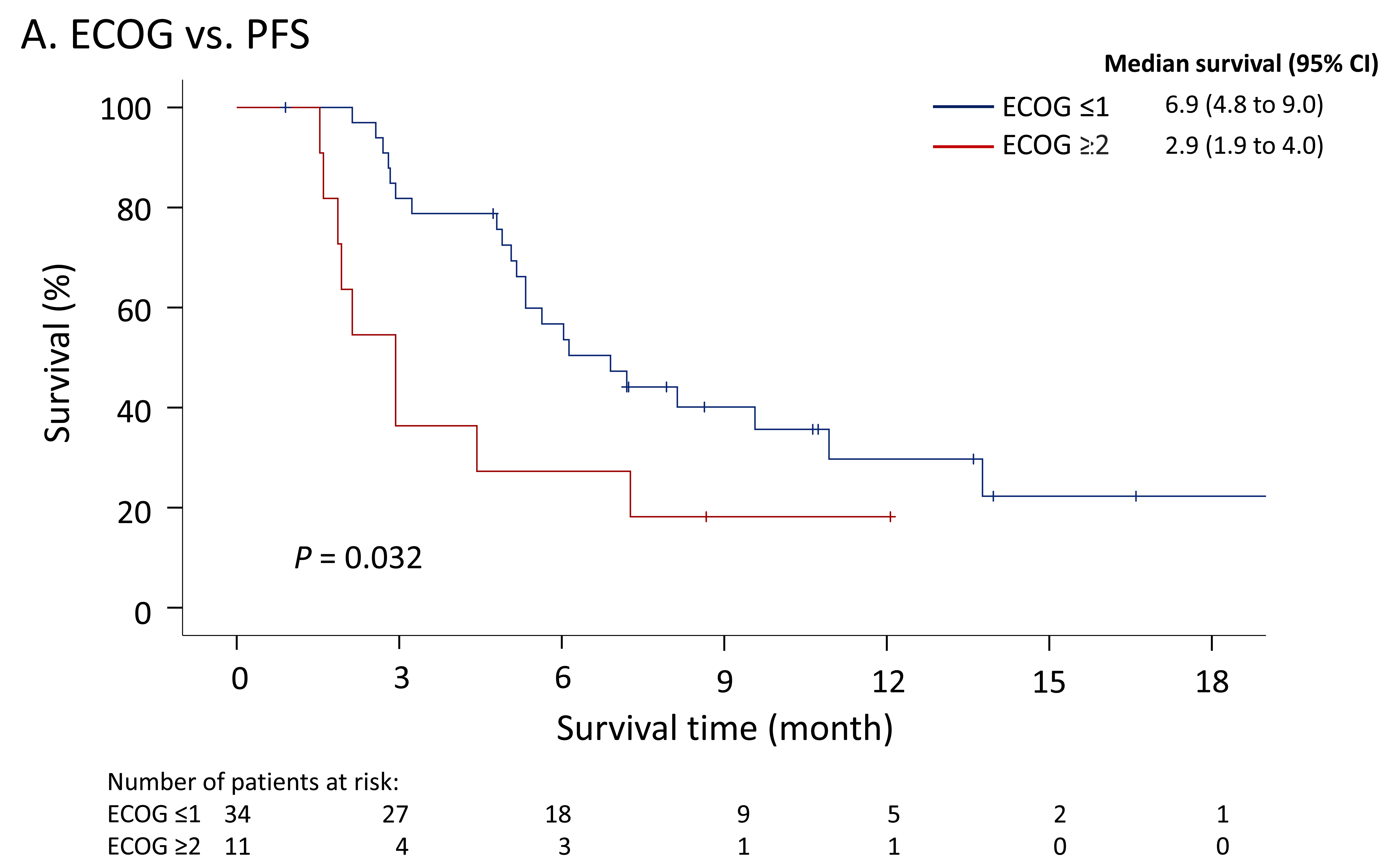

Supplement: oyae306_suppl_Supplementary_Figures [file oyae306_suppl_supplementary_figures.zip › Supplementary Figure/Figure S3A.tif]

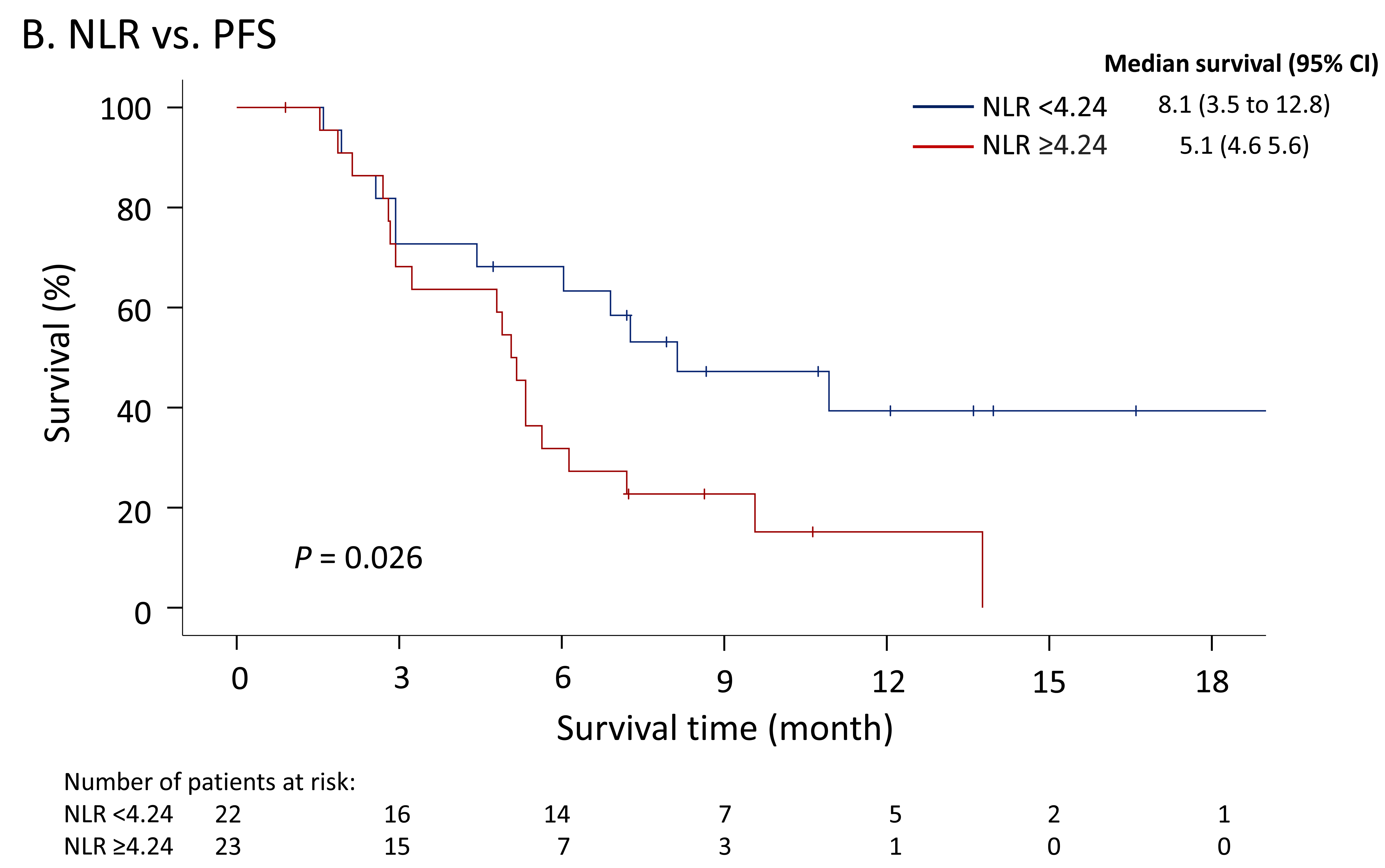

Supplement: oyae306_suppl_Supplementary_Figures [file oyae306_suppl_supplementary_figures.zip › Supplementary Figure/Figure S3B.tif]

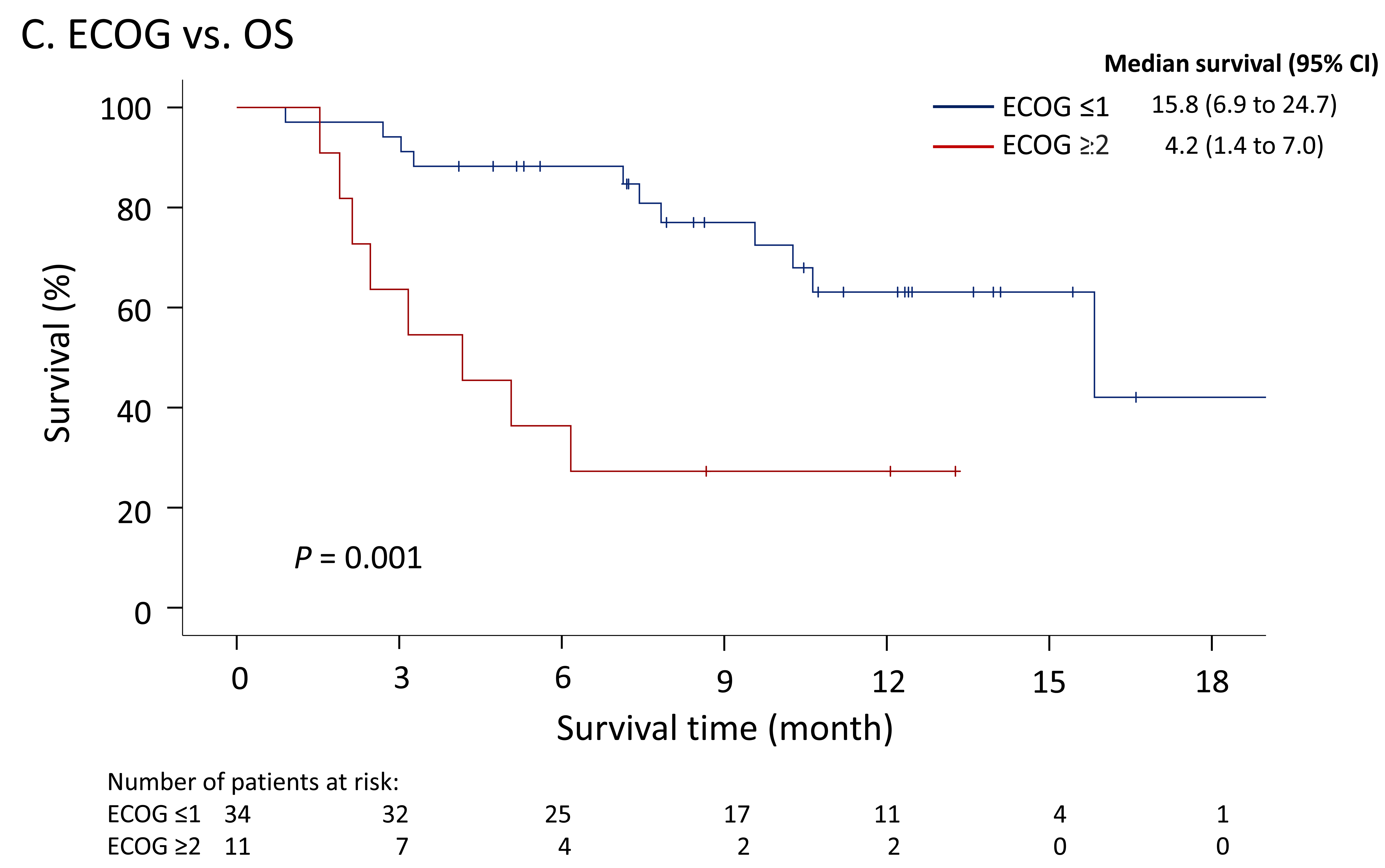

Supplement: oyae306_suppl_Supplementary_Figures [file oyae306_suppl_supplementary_figures.zip › Supplementary Figure/Figure S3C.tif]

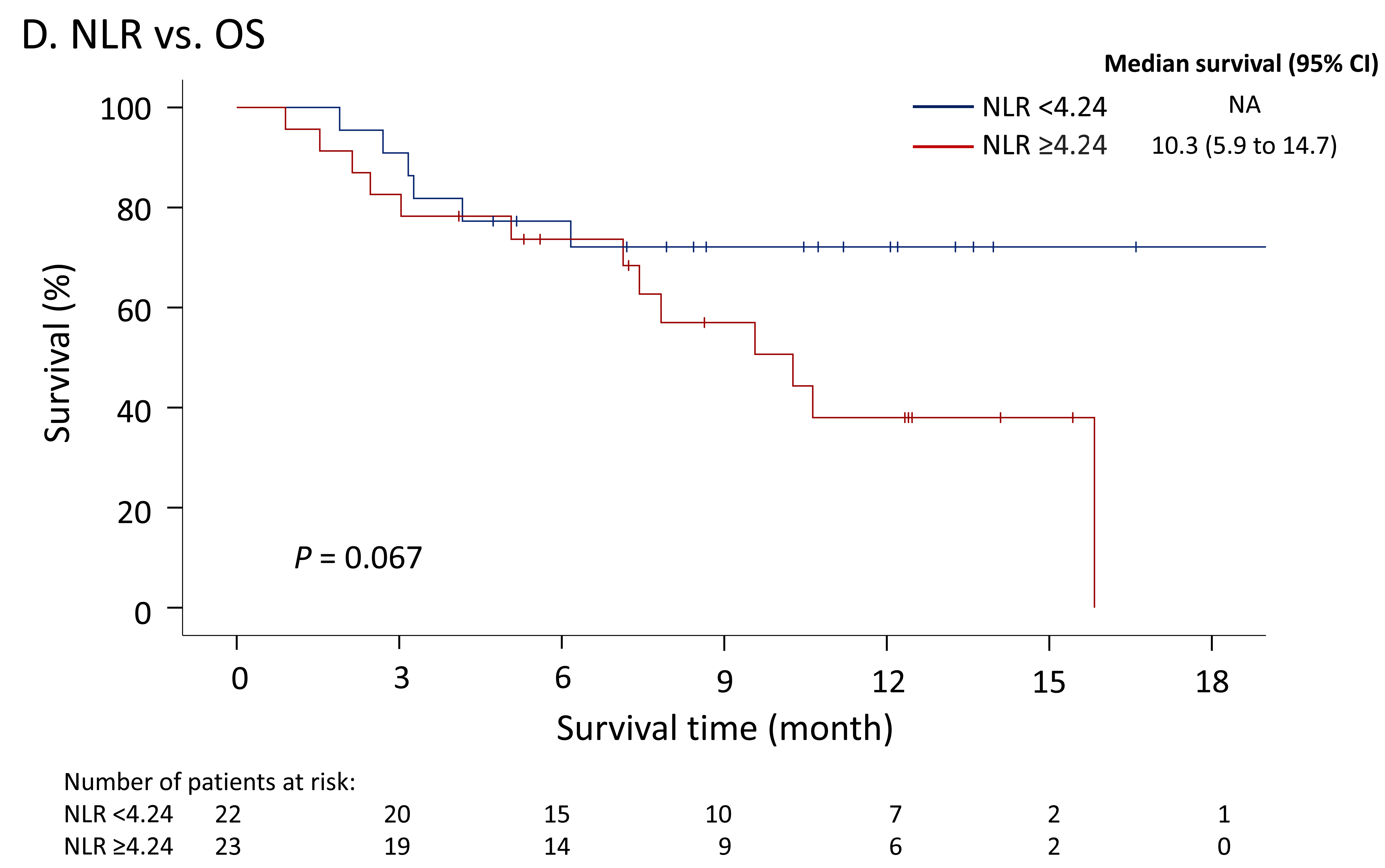

Supplement: oyae306_suppl_Supplementary_Figures [file oyae306_suppl_supplementary_figures.zip › Supplementary Figure/Figure S3D.tif]
